# A perspective on microarrays: current applications, pitfalls, and potential uses

**DOI:** 10.1186/1475-2859-6-4

**Published:** 2007-01-25

**Authors:** Pratik Jaluria, Konstantinos Konstantopoulos, Michael Betenbaugh, Joseph Shiloach

**Affiliations:** 1 Department of Chemical and Biomolecular Engineering, Johns Hopkins University, 221 Maryland Hall, 3400 North Charles Street, Baltimore, MD 21218, USA; 2 National Institute of Diabetes & Digestive & Kidney Diseases, National Institutes of Health, Biotechnology Unit, 9000 Rockville Pike, Building 14A, Room 170, Bethesda, MD 20892, USA

## Abstract

With advances in robotics, computational capabilities, and the fabrication of high quality glass slides coinciding with increased genomic information being available on public databases, microarray technology is increasingly being used in laboratories around the world. In fact, fields as varied as: toxicology, evolutionary biology, drug development and production, disease characterization, diagnostics development, cellular physiology and stress responses, and forensics have benefiting from its use. However, for many researchers not familiar with microarrays, current articles and reviews often address neither the fundamental principles behind the technology nor the proper designing of experiments. Although, microarray technology is relatively simple, conceptually, its practice does require careful planning and detailed understanding of the limitations inherently present. Without these considerations, it can be exceedingly difficult to ascertain valuable information from microarray data. Therefore, this text aims to outline key features in microarray technology, paying particular attention to current applications as outlined in recent publications, experimental design, statistical methods, and potential uses. Furthermore, this review is not meant to be comprehensive, but rather substantive; highlighting important concepts and detailing steps necessary to conduct and interpret microarray experiments. Collectively, the information included in this text will highlight the versatility of microarray technology and provide a glimpse of what the future may hold.

## Review

### Introduction

Although, the principles behind microarray technology were conceived almost 20 years ago and developed from Southern blotting, they did not gain wide spread attention for nearly a decade when researchers were first able to utilize high quality slides with precision robotics resulting in reproducible results [1-3]. For instance, a quick pubmed search with the words, 'microarray and 1995' results in 13 total articles, 5 of which are review articles. Similar searches with the words, 'microarray and 2000' and 'microarray and 2005' result in 288 total articles (78 review articles) and 3906 total articles (1037 review articles), respectively. Despite this relative surge in microarray-related articles, few recent publications address core issues regarding design, implementation, and subsequent data analysis. In covering these and related issues, the present text aims to illustrate the strengths, weaknesses, and application of microarrays, especially to those unfamiliar with the technology.

Today's arrays are vastly superior to their predecessors in terms of quality, probe density, and structural layout [2,3]. Before dealing with these and other characteristics, it is important to discuss, at some length, what microarrays are as well as the fundamental concepts behind the technology. The term microarray is both descriptive and somewhat ambiguous as it is commonly used to describe a variety of platforms including protein microarrays and tissue microarrays [3,4]. A microarray is typically defined as a collection of microscopic spots arranged in an array or grid-like format and attached to a solid surface or membrane, hence the term [4,5]. These spots typically referred to as probes, are designed such that each probe binds a specific nucleic acid sequence corresponding to a particular gene through a process termed hybridization [3]. The sequence bound to a probe, often referred to as the target, is labeled with some kind of detectable molecule or dye such as a fluorophore [4]. The level of binding between a probe and its target is quantified by measuring the fluorescence or signal emitted by the labeling dye when scanned. This signal, in turn, provides a measure of the expression of the specific gene containing the target sequence [2,3].

Although, there are several different types of DNA microarrays, for the purposes of this text only two will be considered; spotted microarrays and oligonucleotide microarrays [1]. Details regarding these two platforms are highlighted in Table 1. Spotted microarrays are often referred to as dual-channel or two-color microarrays because two samples, each labeled with a different fluorophore, are hybridized onto a single slide [3,6]. As a result of combining two samples onto a single slide, only relative expression levels can be determined using spotted arrays [1]. The probes in spotted arrays are oligonucleotides, complementary DNA (cDNA), or fragments of polymerase chain reaction (PCR) products; each type conferring different properties to the spotted array. Despite these differences, all spotted arrays are similar in terms of: array construction, target preparation, and data analysis [2,7]. In contrast, oligonucleotide microarrays also referred to as single-channel microarrays are hybridized with only one sample and therefore generate absolute expression levels. These arrays utilize probes designed to complement mRNA sequences and are produced using various methods including *in situ *synthesis, some type of deposition method, or photolithography [3,4].

**Table 1:** Comparison of cDNA and oligonucleotide microarrays

**Feature**	**Oligonucleotide microarrays**	**Spotted microarrays**
Typical probe length	18 – 30 mers	500 – 1000 base pairs (cDNA) 25 – 100 mers (oligo)
Spot density	> 500,000 features per slide	≤ 80,000 spots per slide
Hybridization Specificity	High specificity	Not as specific with possible cross-hybridization
Expression levels	Normalized for single-channel system	Ratio-based normalization for dual-channel system
Advantages	• Large-scale production • Highly reproducible • Detection of alternative splice variants • Precise measurements • Information can still be generated for genes without expression in the reference/control sample	• Lower costs • Signal amplification is not needed • Independent of genome sequence • Elimination of artifacts from spotting
Disadvantages	• Expensive • Few producers of necessary equipment and buffers • Difficulty detecting low abundance transcripts	• Hybridization is dependent upon length of sequences spotted • Labeling efficiency of dyes is an issue • Little information is generated for genes without expression in the reference/control sample • Handling of clones

As alluded to earlier, two important elements of microarray technology are target preparation and probe construction. Depending on the type of microarray being used, different cellular components can be used for target generation including: RNA, genomic DNA, cDNA, complementary RNA (cRNA), and PCR products [6,7]. Regardless of which of these are used, ensuring the quality, stability, and reproducibility of the generated targets is paramount for subsequent processing. Similarly, probes can consist of any of the following: cDNA, oligonucleotides, fragments of PCR products, restriction-enzyme digested fragments, oligomers, or expressed sequence tags (ESTs) [1,2,6]. Irrespective of the exact composition of the probes, they all serve the same basic function; binding very specific sequences. Although, probes are constructed in a variety of ways, depending on the type of array and the specific application, the same public databases are referenced for sequencing information [2,5,7]. Typically, arrays are fabricated with duplicates of each probe, enhancing the likelihood of observing hybridization for each gene.

**Figure 1:**
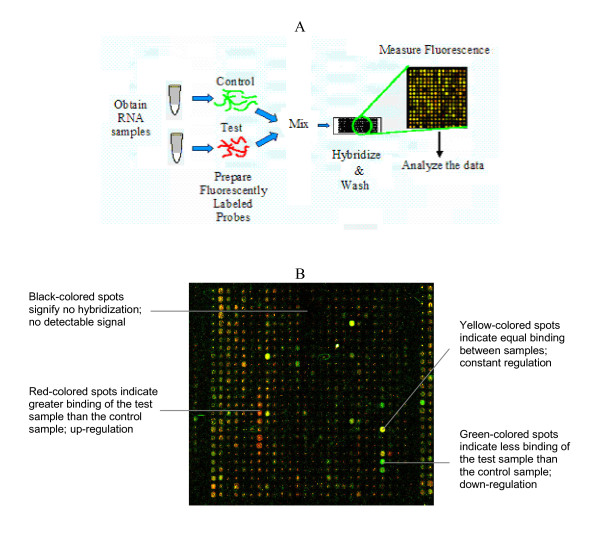
(A) Schematic highlighting the major steps in a cDNA microarray experiment [8]. Briefly, RNA from biological samples is isolated and checked for purity. Of the two RNA samples shown, one is considered the 'test' and the other the 'control'. Each sample is labeled with a different fluorescent dye, indicated by the green and red colors. The two samples are then mixed and hybridized onto a spotted microarray slide. Once the slide is washed, it is scanned at two different wavelengths, each corresponding to one of the dyes. (B) Image of scanned microarray wherein a number of distinct colors can be seen. Each color is representative of a certain amount of binding. For the image shown, the test sample was labeled with Cy5 and the control sample was labeled with Cy3.

A simple schematic of the entire process for a spotted cDNA microarray experiment can be seen in Figure 1A[8]. Briefly, total RNA, once isolated from a sample, is reverse transcribed to produce cDNA, labeled with fluorescent dyes, and then hybridized onto the spotted array [5,8]. Hybridization is quantified using the intensities of the fluorescent dyes at particular wavelengths. By comparing fluorescence intensities, genes that are differentially expressed between the two samples can be identified, along with the direction of that difference (i.e. over-expression or under-expression relative to a control) [2,3]. For example, Figure 1B illustrates the significance of each color when the test sample is labeled with Cy5 and the control sample is labeled with Cy3. In this case, black represents no binding (i.e. no signal), green indicates greater binding of the control sample than of the test sample referred to as down-regulation, yellow indicates equal binding between the two samples, and red indicates greater binding of the test sample than of the control sample referred to as up-regulation. If the dyes are used in reverse (i.e. Cy5 is used to label the control sample and Cy3 is used to label the test sample) the colors would have the opposite representations [3,7].

### Biological Systems

Although a multitude of microarrays are commercially available, each designed for a specific species or general family of organisms; these arrays are limited by the information available in genomics databases [2,9]. Though, the genomes of only a few species have been entirely sequenced and made available to the public, microarrays for a large number of species are available [10,11]. For instance, checking the website for Affymetrix reveals genome-wide arrays are available for the following microbes: *Bacillus subtilis*, *Escherichia coli*, *Pseudomonas aeruginosa*, members of the genus *Plasmodium*, *Staphylococcus aureus*, and members of the genus *Saccharomyces*.

Small, custom arrays can be designed for many more species as long as genomic sequences are available for a particular organism or family of organisms [2,9,11]. Continued genome exploration has resulted in the need for frequent updating and re-organization of spotted arrays. With more information constantly coming online, microarrays are continually refined to enhance reproducibility and detection levels of weak signals by modifying the positioning and sequences of the ESTs spotted [2,10]. As previously mentioned, ESTs are essentially unique segments of cDNA identical to a portion of a gene, thereby acting as binding domain. In addition, valuable information can still be ascertained by hybridizing samples onto arrays designed for other species [12,13]. So, even without an entire genome being spotted onto commercially available arrays for a given species, microarray experiments can still yield important results.

### Limitations, Pitfalls, and Design Considerations

Any discussion regarding microarray technology would be incomplete without a detailed examination of the various limitations and complexities inherently present. Such a discussion is vital to properly conduct microarray experiments and analyze microarray data; overcoming technological limitations in the process. Before conducting microarray experiments, the following questions need to be addressed: what are the goals of the experiment, what biological comparisons are most relevant to these goals, how should the experiments be designed and performed taking into account the various sources of variability, which platform should be used, what controls need to be in place, and how can the results be verified [14,15]. In approaching these and other relevant questions, a great deal of information regarding microarray technology can be ascertained.

To answer the first two questions regarding goals and relevant comparisons, a number of resources can be referenced. Several organizations such as the Microarray Gene Expression Data (MGED) Society and the European Bioinformatics Institute (EBI) have established guidelines to aid researchers in the design and implementation of microarray experiments [8,9,16]. In general, narrowing the objectives of a microarray study can provide insight into which biological samples should be compared. Clear and concise goals also help define the scope of the study, providing a framework within which subsequent experiments can be proposed and implemented. One of the most commonly sited proposals is the Minimum Information About a Microarray Experiment (MIAME) that includes a series of recommendations and standards on collecting and analyzing microarray data [16,17]. This document was designed to allow data generated by microarray experiments to be interpreted and reproduced with certainty. In addition, repositories such as the Gene Expression Omnibus (GEO) created by the National Center for Biotechnology Information (NCBI) and ArrayExpress created by the EBI have been established to store and share gene expression data [16,17].

Microarray experiments are typically constructed using one of several different design layouts including loop, reference, and saturated [17]. Each of these designs specifies the number of samples needed and the manner in which samples should be compared in order to obtain a desired level of accuracy and reproducibility. The loop design is relatively simple and involves minimizing the number of duplicates while retaining pertinent comparisons. This setup can be problematic because failure of a single array can greatly magnify error and statistical variance [17]. Another scheme is the reference design in which a common reference sample is used with each hybridized array. This system allows any array to be compared to any other array even extending to other experiments as long as the same reference sample is used [16,17]. However, this setup can become costly if the goals of the experiment require multiple comparisons to be made. In contrast, the saturated design involves making every possible comparison exactly once [17]. This approach is balanced and simple to establish, however, it is not applicable to all conditions and is not appropriate when a series of experiments are planned. Ultimately, the design selected must address the goals and requirements of the experiment being conducted. Without these and other considerations, errors in analysis including the identification of false positives can result, masking underlying patterns and incorrectly deciphering biological behavior.

There are multiple sources of variability such as differences in: arrays, dye labeling, efficiency in reverse transcription, and hybridization [10,14]. Some of these issues relate back to the actual production of arrays and how probes are prepared; elements of quality control on the part of the manufacturer. The remaining issues are best overcome by: incorporating replicates to generate statistical significance (i.e. averages and variance), performing dye-swapping experiments, and pooling samples to minimize biological variation [6,7,14]. Both technical and biological replicates are commonly employed, each with a different purpose in mind. Technical replicates aim to quantify procedural variations such as sample preparation and handling [16]. In contrast, biological replicates aim to identify variation in the biological system being studied [16]. Similarly, dye swapping involves switching the dyes used for labeling in a manner that prevents one type of sample from being labeled by a single dye. This setup helps account for the dye effect; an important systematic error that stems from differences in the properties of the dyes. The pooling of samples also reduces inherent variation in biological samples while at the same time generating sufficient sample quantities for subsequent processing [17].

As discussed earlier, there are two main platforms to consider when designing microarray experiments; spotted microarrays and oligonucleotide microarrays. The advantages and disadvantages of each are outlined in Table 1 along with examples of when a particular platform is most beneficial [2,3]. For example, oligonucleotide microarrays are ideal for time-course experiments because each array is hybridized with only one sample, allowing any array to be compared to any other array. This translates into requiring a smaller number of total samples for the same number of duplicates while at the same time more accurately representing the control for a given condition. Similarly, for static conditions in which a basic comparison between treated and untreated cell populations is needed, dual-channel microarrays may be the best fit. Each application has its own set of criteria that should be carefully evaluated to determine the best platform to use [1,15]. For instance, if specific genes are to be investigated, it should be verified that the platform includes those particular genes with the desired number of replicates. A simple search online will reveal a multitude of companies that manufacture microarrays and allow customers to construct their own custom arrays using specialized software.

In addressing the various sources of error, systematic or otherwise, proper controls need to be implemented. There are two types of controls, as they pertain to microarray technology; internal controls and external controls [1,18]. Internal controls check for the quality of the printed microarray whereas external controls account for performance in terms of sensitivity and robustness. The internal controls often used include: hybridization controls, poly-A controls, normalization control sets, and housekeeping genes [10,18]. Each type of control is commonly found in commercially available arrays and serves a distinct function relating to one specific aspect of microarray processing. In addition, samples can be spiked with particular agents to isolate or quantify detection limits, non-specific noise, and similar parameters [17].

Similarly, a number of approaches can be taken to minimize external variables such as discrepancies in: growing and preparing biological samples, isolating and purifying RNA, cell synchronization, hybridization protocols, and target preparation [14,19]. In general, standardizing procedures can greatly reduce these errors introduced during the course of the experiment. Although, the preparation of control samples used in a microarray experiment is typically not critical, the samples must be stable throughout the experiment and be reproducible. To verify the quality of purified RNA and/or cDNA gel electrophoresis and/or spectrophotometry should be used. With regards to cell synchronization, whole-culture methods such as serum starvation (a method in which cells are deprived of animal serum, a commonly used media supplement, to direct cells towards quiescence) and DNA arrest (a general method of using chemical or pharmacological agents to prevent one or more phases of DNA replication, suspending cells in a particular stage of the cell cycle) are typically used [20,21]. However, selective methods such as mitotic shake-off, a method that involves shaking a flask or plate to remove cells undergoing mitosis because these cells are loosely attached, have also been used due to questions about the validity of whole-culture methods [20,21]. Whatever synchronization method is used should be applied to all of the biological samples to ensure a valid comparison is being made.

**Figure 2:**
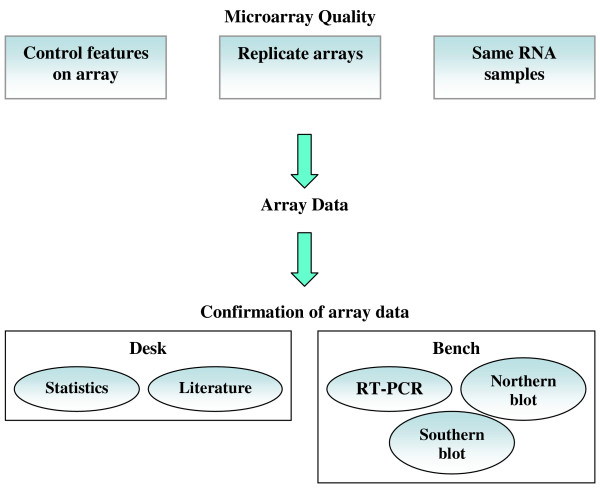
Common steps employed to ensure quality and validity of microarray results. From a quality control standpoint, replicates should be performed using RNA samples prepared at the same time under the same conditions. Various features of the arrays being used should also be known, especially the controls. To verify the results generated from microarray experiments, a combinatorial approach is usually needed; checking the statistical significance associated with the expression levels of specific genes, reviewing the literature, and conducting additional experiments such as RT-PCR or Northern blotting.

Typically to validate microarray results any one of a number of techniques such as RT-PCR, Northern blotting, Western blotting, and even the use of multiple microarray platforms can be employed [10,14,18]. Figure 2 illustrates which of these methods are relevant to which aspects of microarray analysis. As mentioned earlier, verification is critical in order to assign distinct expression patterns to specific genes with certainty (i.e. statistical significance) because of the inherent variability present in microarray data. Since a single microarray experiment evaluates the expression levels of tens of thousands of genes simultaneously, it would be extremely impractical to verify each and every gene using any of the methods listed above. Instead, what is typically done is that a number of key genes are verified depending on the purpose and scope of the experiment [2,5]. In addition, not every gene can be assayed using each verification method because the necessary components may not be available such as monoclonal antibodies necessary for Western blotting or labeled primers for RT-PCR. As a result, multiple methods are often used to verify the results of microarray experiments.

### Normalization and Statistical Methods

As described previously, expression levels for a given gene are determined using intensity values. One distinction between dual-channel microarrays and single-channel microarrays is that the former generates relative expression levels whereas the latter generates absolute expression levels [1,6]. This distinction stems from the fact that dual-channel microarrays are hybridized with two different samples; one considered the test sample and the other considered the control sample. As a result, the expression level determined for a specific spot or gene is dependent upon both samples and is a ratio of the form:

Expression level=test sample intensitycontrol sample intensity
 MathType@MTEF@5@5@+=feaafiart1ev1aaatCvAUfKttLearuWrP9MDH5MBPbIqV92AaeXatLxBI9gBaebbnrfifHhDYfgasaacH8akY=wiFfYdH8Gipec8Eeeu0xXdbba9frFj0=OqFfea0dXdd9vqai=hGuQ8kuc9pgc9s8qqaq=dirpe0xb9q8qiLsFr0=vr0=vr0dc8meaabaqaciaacaGaaeqabaqabeGadaaakeaacqqGfbqrcqqG4baEcqqGWbaCcqqGYbGCcqqGLbqzcqqGZbWCcqqGZbWCcqqGPbqAcqqGVbWBcqqGUbGBcqqGGaaicqqGSbaBcqqGLbqzcqqG2bGDcqqGLbqzcqqGSbaBcqGH9aqpdaWcaaqaaiabbsha0jabbwgaLjabbohaZjabbsha0jabbccaGiabbohaZjabbggaHjabb2gaTjabbchaWjabbYgaSjabbwgaLjabbccaGiabbMgaPjabb6gaUjabbsha0jabbwgaLjabb6gaUjabbohaZjabbMgaPjabbsha0jabbMha5bqaaiabbogaJjabb+gaVjabb6gaUjabbsha0jabbkhaYjabb+gaVjabbYgaSjabbccaGiabbohaZjabbggaHjabb2gaTjabbchaWjabbYgaSjabbwgaLjabbccaGiabbMgaPjabb6gaUjabbsha0jabbwgaLjabb6gaUjabbohaZjabbMgaPjabbsha0jabbMha5baaaaa@7EE3@

Therefore, the expression level for a gene in a dual-channel microarray is relative, not absolute. In contrast, single-channel arrays are hybridized with only one sample and therefore the expression level for a given gene is absolute [2,4].

Once a scanned image for a hybridized microarray has been generated, visual inspection of the data can proceed, prior to normalization. This entails using imaging software to exclude specific spots with poor signaling and adjust the size/shape of grids that encompass the spots [10,22]. Next, normalization procedures can be applied to the data. Essentially, normalization accounts for differences in labeling efficiencies and detection levels for the fluorescent dyes as well as differences in the quantity/quality of RNA samples [4,10,23]. As such, normalization can be thought of as the first level of filtering applied to the data. Advanced statistical software packages offered by companies such as Partek and Acuity are commonly used. Private research institutes such as The Institute for Genomic Research (TIGR) and The Sanger Institute along with academic facilities around the world also provide free software for microarray analysis [22].

**Figure 3:**
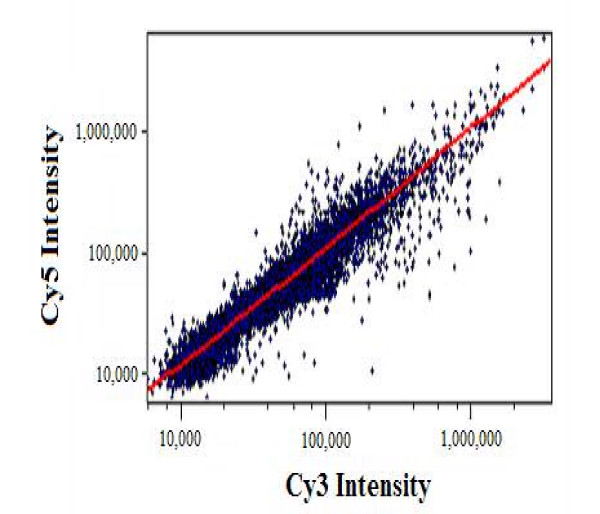
Scatter plot of measured intensities for both fluorescent dyes on a log-log scale prior to normalization. The measured intensities are in arbitrary units. Each point in the graph represents a single spot on a hybridized microarray. In addition, the red line shown is the best-fit line calculated for the data with a slope that is close to unity.

Although a number of normalization techniques can be applied to microarray data, the most commonly used are: total intensity, regression, and ratio statistics [18,23,24]. All three of these techniques assume that for some group of genes on the array, the average expression ratio is equal to one [10]. Total intensity normalization assumes both samples (test and control) are comprised of the same amount of RNA and the total amount of RNA hybridized from each sample is the same. Therefore, the total intensity calculated from all the spots on an array should be the same for both fluorescent dyes (channels) [5,10]. Conversely, normalization using regression presumes that a significant number of genes are expressed to the same extent in both samples; a reasonable assumption for samples that are fairly similar [22]. If the labeling and detection efficiencies for the two samples were equivalent, then the slope of the plot shown in Figure 3 would be one [10,22]. Figure 3 was constructed from unnormalized data obtained from a single, spotted cDNA array. Two different samples were hybridized onto the array, each labeled with a different dye. The graph illustrates inherent differences between the dyes in terms of labeling and detection efficiencies due to the characteristics of each dye such as stability. Using regression techniques, the best-fit slope is calculated and modified to be equal to one by adjusting gene intensities. Lastly, normalization using ratio statistics assumes that there exists some subset of genes with the same expression levels in both samples [10,23]. These housekeeping genes, as they are often referred to, are used to calculate probability densities which in turn allow the mean expression ratio to be adjusted to one. Each of these techniques calculates a normalization factor that is then used to scale the data, accounting for the variations previously mentioned [4,9,10].

Following normalization the data can be probed using a host of statistical techniques that evaluate and ultimately decipher microarray data. For the purposes of this text, only two types will be touched upon briefly; clustering and hypothesis testing [6]. In general, both types of statistical methods strive to categorize, shape, and illuminate underlying patterns and therefore can be very useful in analyzing microarray data [23,25]. However, both methods rely on different underlying principles and assumptions that directly influence their employment.

**Figure 4:**
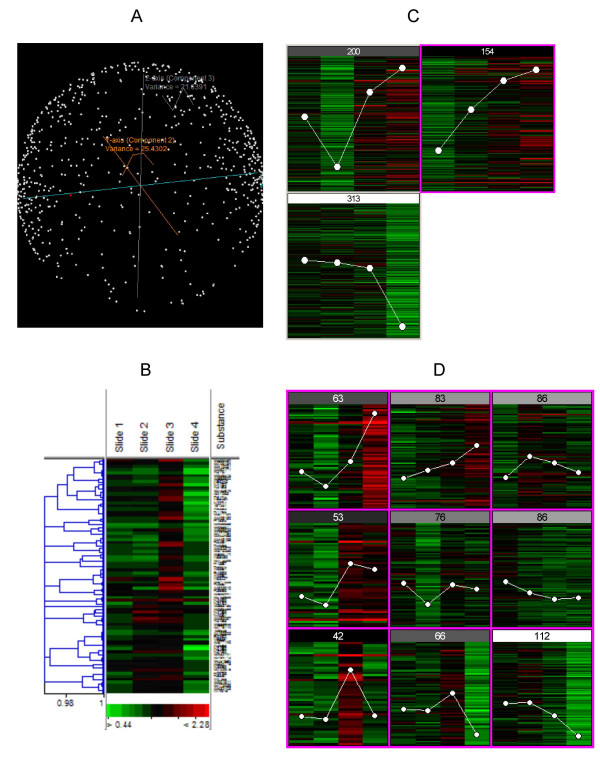
Visualizations generated from the following clustering algorithms: (A) Principle component analysis (PCA), (B) Hierarchical, (C) K-means, (D) Self-organizing maps (SOMs). Each image depicts the results of a specific clustering algorithm applied to the same set of genes, approximately 700 genes. The data shown was generated from cDNA microarrays hybridized with human cells grown under varying conditions. Each algorithm segregates the data differently, based on specific parameters and conditions.

Clustering algorithms rely on calculating some kind of 'distance metric' to position gene expression levels into a matrix of sorts with a certain level of commonality [10]. Both differentially expressed genes and groups of genes with similar expression patterns can be highlighted using clustering techniques. The most widely used clustering algorithms include: hierarchical, self-organizing maps (SOMs), k-means, and principle component analysis (PCA) [8,10,18]. The mathematical formulations behind each of these methods are too complex and lengthy to be dealt with here, and so for the sake of brevity, very basic information will be covered in this text with a strong recommendation to consult specific references [10,18,23]. Typically, these and other algorithms are used to create a more accurate and meaningful interpretation of the data. Figure 4 illustrates how four different algorithms (when applied to the same data set) can generate vastly different groupings; each providing a different perspective on patterns present in the data. The data shown in Figure 4 was obtained by hybridizing human cell lines grown under varying conditions onto cDNA microarrays. By applying clustering algorithms in sequence, one after another, synergy is possible, lessening the shortcomings in each individual method. For example, it is common to apply PCA to data prior to analysis with either k-means clustering or SOMs in order to generate an estimate for the number of clusters to be formed [10].

Hierarchical clustering, quite possibly the most commonly used clustering algorithm, links every gene in an array to every other gene through a series of expanding brackets that collectively form a dendrogram [22]. Genes deemed to be closely associated, referring back to the concept of a distance metric which in fact can be computed using several different statistical frameworks, are connected by a node [18,23]. Each node links to other nodes of various sizes, in a repetitive process until every possible pair of genes are linked, as illustrated in Figure 4B. This type of clustering is popular due to its simplicity and ability to visualize the data. However, the statistical framework has several disadvantages including: not being able to account for multiple ways in which expression patterns can be similar, having difficulty assimilating large quantities of data, and forcing a hierarchical system upon a data set that does not truly exhibit a hierarchical lineage [10,23].

Unlike hierarchical clustering, SOMs require initialization and are much less rigid in terms of structure while at the same time remaining robust and unique. Initialization involves defining a particular geometry, typically a grid or ring, with a specified number of groups or nodes [10,23]. These nodes are mapped into a high dimensional space and successive iterations, usually tens of thousands, look to reduce the number of dimensions [10]. The algorithm also makes use of weighted vectors to select and group similar data entries together, essentially training itself after each phase. The end result of this process is a self-organized network that can be visualized [4,6]. These and other features make SOMs a powerful tool in exploratory studies with an emphasis on visualization.

Similarly, k-means clustering aims to partition gene expression data into a specified number of disjoint clusters. Again, a distance metric is used in these calculations and can be specified by the user. Genes within a cluster are deemed similar to one another, but clusters are deemed dissimilar to one another producing a series of clusters that are not related or connected, opposite of the structure produced in hierarchical clustering [7,10]. Essentially, each gene is placed in one of the clusters initially specified and distances between clusters are calculated. Next, genes are moved from one cluster to another until a local stability is reached in which the distance between clusters is maximized while at the same time minimizing the distance between members of a given cluster [22,23]. This method is reliable and relatively simple and therefore is useful in analyzing data for which there is some prior knowledge such as classifying serotypes or strains. Ensuring that the partitions constructed using k-means clustering have some type of real or actual significance is where the difficulty lies [10].

PCA is an algorithm that relies on visually highlighting similarities in data in a manner that reduces the number of dimensions. It can be applied to any number of data sets from a small group of genes within a single array to groups of experiments each with a number of arrays [10,22,23]. As seen in Figure 4A, the plot generated from PCA allows patterns in data to be visualized by examining the proximity of clusters. The method implements a series of calculations to best separate the data and project that final analysis onto a 2 or 3 dimensional plot [22,23]. When combined with other clustering methods, PCA can be a very useful tool, as described earlier.

Besides clustering algorithms another statistical approach typically used to analyze microarray data is hypothesis testing which aims to establish statistical significance associated with divergent findings. If a group of genes, perhaps genes that constitute a particular pathway, are differentially expressed between two samples, hypothesis testing can quantify the extent of those differences. Hypothesis testing is comprised of the following steps: specify the null hypothesis and the alternative hypothesis, select a significance level, calculate a statistic analogous to the parameter designated in the null hypothesis, calculate the probability value (p-value), compare the p-value with the significance level, and finally accept or reject the null hypothesis [7,26]. At the end of these steps, an observed outcome is associated with a statistical likelihood indicating whether or not the observed outcome is the result of chance and not some real difference or phenomenon [26]. Application of hypothesis testing is most useful when evaluating microarray data with specific genes or groups of genes in mind as opposed to discovery or exploration.

### Current Uses

A large number of microarray-related studies in the past have aimed to either characterize diseased cells in comparison to healthy cells or highlight the genes involved in a particular biological pathway [4,8]. Infrequently, studies were undertaken for other purposes such as gene discovery or examining distinct cellular properties [5,6]. However, in recent years, the number of studies utilizing microarrays in some capacity has increased greatly. More and more studies are relying on microarrays to provide insight into observed physiology, essentially using microarrays to further characterize biological systems [3,9]. In most of these cases, microarray analysis has generated interesting results, but also raised additional questions requiring further investigation, limiting its successful implementation.

For instance, the application of bio-informatics tools such as microarrays to characterize microbial populations exposed to toxins and pollutants has been explored [27]. Being able to understand the catabolism of xenobiotics could enhance bioremediation processes with a direct impact on pollution control and environmental organization [27]. In addition, the exploration of previously uncharacterized microbes using microarrays could identify novel genes with relevant functionality [27]. In this context, a number of studies have focused on specific issues such as investigating how *Candida albicans*, a human fungal pathogen, is able to protect itself from the toxic effects of nitric oxide produced by the immune system [27,28]. Microarray analysis revealed a group of nine genes were over-expressed during exposure to nitric oxide. Of these nine genes *yhb1*, which produces a flavohemoglobin that detoxifies nitric oxide, was the most highly expressed [28].

Evolutionary studies using microarrays have also gained prominence with the use of species-specific arrays in parallel. For example, researchers hybridized DNA from the progeny of two yeast strains, one with a particular evolved trait (i.e. mating discrimination) and the other without, onto oligonucleotide microarrays [29]. The arrays used in this study were designed to detect a multitude of polymorphisms between the two strains. Adaptive mutations were identified by linking polymorphisms to the evolved parental strain [29]. Investigators then mapped known genes and constructed a computer simulation capable of evaluating various parameters impacting mapping precision [29]. Finally, the researchers applied their method to yeast strains adapting to a changing glucose-galactose feed illustrating mutations in the same gene can lead to parallel adaptation [29]. Similarly, scientists compared community-acquired invasive *Staphylococcus aureus *strains to isolates from healthy people using microarray constructed from 7 previous sequencing projects [30]. Ten dominant lineages were identified; each with a distinct group of genes with potential functions related to virulence and resistence. Subsequent analysis suggested a common ancestor could be traced back for all of the strains studied, but evolutionary divergence must have occurred early on [30].

The development of therapeutics has also benefited from the implementation of microarrays as evidenced by a number of recent publications. For example, scientists examined gene expression profiles from patients with chronic drug abuse, intending to better understand addiction and therefore formulate better treatments [31]. Analysis of the array data revealed very little overlap in the expression patterns for heroin and cocaine users [31]. These findings were contrary to widely held views regarding the shared effects of heroin and cocaine on dopamine, thus prompting reassessment of previous assumptions [31]. Another study, examined the mechanism behind acquired nisin resistance in bacteria [32]. Researchers found genes involved in the following pathways to be expressed differentially between resistant and non-resistant *Lactococcus lactis *strains: cell wall biosynthesis, energy metabolism, fatty acid and phospholipid metabolism, regulatory functions, and metal/peptide transport and binding [32]. Using this information, the researchers established mutant strains that either had genes knocked down or over-expressed and found these mutants had varying levels of nisin resistance as compared to the parental, wild-type strains [32].

In terms of disease characterization and detection, microarrays are also finding use. For instance, the pathogenicity of coxasackievirus B3 (CVB3) was examined; in humans this virus adversely affects the heart muscle [33]. Using cDNA microarrays, researchers compared murine hearts infected with the virus against non-infected murine hearts. In addition, oligonucleotide microarrays were used to compare infected HeLa cells over time [33]. Together, these experiments identified a number of differentially expressed genes, providing clues as to the precise sequence of events following infection. Similarly, the use of custom microarrays to characterize unknown samples from water treatment centers as part of a quality control measure was examined [34]. The microarray was constructed to target 16S ribosomal RNA (rRNA) from several groups of nitrifying bacteria and tested against reference samples with some success [34].

Using microarrays in the capacity of diagnostics has also become relatively popular especially in the context of outbreaks for which rapid diagnostic tools are needed to quickly evaluate pathogens and identify specific strains or serotypes [9,35,36]. For example, a microarray was constructed specifically to probe single nucleotide polymorphisms (SNPs) for foot and mouth disease virus (FMDV) [37]. The results were classified using statistical methods in order to develop a procedure to test for specificity with diagnostic application [37]. Similarly, a study combined the use of microarrays with reverse transcription-PCR to differentiate between two genetically similar enteroviruses; enterovirus 71 (EV71) and coxsackievirus A16 (CA16) [38]. This approach had a diagnostic accuracy of at least 92% for each of the two viruses as compared to reverse transcription-polymerase chain reaction (RT-PCR) and neutralization testing [38]. Currently, studies are being conducted to explore the feasibility and implementation of similar methods for other pathogens [38,39].

### Potential Uses

With advancements in software and robotics technology, microarrays are becoming inexpensive, robust, and reliable [2,5]. The availability of custom arrays designed to probe a small subset of genes (usually several hundred) or specific pathways have also enhanced the potential utilization of microarray technology [1,2,9]. This section was designed to highlight the latest advances in the technology, speculate on novel applications of microarray technology, and outline areas of research that have just begun to use microarrays. Together these aspects portray the potential of microarrays in terms of applications as well as from a technical standpoint.

Breakthroughs in various aspects of the technology from fabrication to commercialization are continually influencing the kinds of microarrays and techniques researchers are using. Currently, microarray experiments are conducted in a series of steps with each step being distinct and in a particular order. However, newly developed chips equipped with electronic circuitry are circumventing a number of these steps particularly sample labeling [8,9]. In addition, a number of companies and research facilities now offer specialized arrays for detection, sequencing, and/or diagnostic purposes [3,6]. By commercializing such highly specific arrays, data gathering is being expedited for studies with explicit purposes. An integrated platform like the lab-on-a-chip (a system that combines multiple manipulations including sample mixing, labeling, and separation onto a single chip) is also influencing microarray technology. The miniaturization and automated techniques used to construct the lab-on-a-chip system are being applied to microarrays leading to arrays that can be readily used for high-throughput applications [7].

One of the most promising areas of research includes classification; particularly in the context of diseases and/or pathogens [40-43]. For instance, in 2002 researchers at the National Cancer Institute used microarrays to organize biopsy samples of diffuse large-B-cell lymphoma from more than 200 patients [44]. They identified 3 subgroups with varying expression of 17 distinct genes; constructing a model capable of predicting survival rates following chemotherapy [44]. In another study, researchers used microarrays to confirm previous classifications of nonpathogenic, low-pathogenic, and high-pathogenic types for 94 different *Yersinia enterocolitica *strains [45]. Researchers identified clusters of genes as being representative of each type (i.e. being present in one group, but not in another) with functional implications [45].

Another arena in which microarrays may prove beneficial is discovery; primarily in the context of gene functions and the identification of novel organisms. For instance, in a recent study researchers analyzed an *Escherichia coli *strain, A49, with a mutation in the *rnpA *gene making it sensitive to temperature and therefore unable to grow at or above 43°C [46]. Under varying growth conditions, researchers found a number of genes differentially expressed. Careful review of these genes revealed RNase P, the mutated gene product, may have more functions than what had been proposed previously, especially in the context of handling precursor RNAs [46]. Researchers in 2003 constructed a custom array with highly conserved arrangements from every fully sequenced viral genome available in GenBank [39]. Next, they hybridized a viral isolate from a severe acute respiratory syndrome (SARS) patient onto the array and found a previously unidentified coronavirus [39]. Subsequent work involving viral sequencing verified these findings and showcased the potential of custom arrays to expedite the identification of pathogens; a virtual necessity in combating future outbreaks [39].

In terms of biological products, particularly vaccines and therapeutic proteins, microarrays may also find use. As detailed in various governmental regulations, slight variations in a biological process may result in distinct final products; requiring further testing and validation [9]. Microarrays may very well provide a means of avoiding these procedures by establishing criteria (i.e. expression patterns for a small set of genes) that can be used to verify consistency and reproducibility. Extensive research would, however, be required to first establish the necessary criteria. In addition, it should be stressed that in this particular application, microarray results would have to be viewed in terms of patterns for a group of genes rather than the expression levels of individual genes [9,15]. This is because the variability associated with a single gene can exceed levels needed to verify or validate biological processes, whereas the variability in groups of genes where an overall pattern is decoded is much less [10].

Another area that has and continues to find microarray technology beneficial is pathway probing; illumination of biological pathways. Often, microarray data alone cannot decipher the sequential steps necessary for a particular mechanism to occur, however, it can provide insight into what genes or groups of genes should be investigated further [15,35]. For instance, a paper published in 2003 used microarrays together with other experimental techniques to decipher a pathway responsible for regulating the expression of cyclooxygenase-2 (COX-2), a pro-inflammatory protein associated with arthritis and pain [47]. A continuation of this work was published in 2005 further illuminating the pathway and possible feedback mechanisms with important therapeutic implications [48].

Perhaps the greatest potential lies in combining two fields within the scope of bio-informatics; genomics and proteomics. Genomics is the study of genes and their function whereas proteomics is the study of proteins and their functions [42,49]. By utilizing tools from each of these two disciplines, researchers may be able to construct more accurate and comprehensive models depicting specific biological processes. For example, in a recent study both two-dimensional gel electrophoresis and microarrays were used to identify genes involved in the acclimation of changing visible light in cyanobacteria [50]. Focusing on the organism *Fremyella diplosiphon*, researchers found approximately 80 proteins with different levels between cells grown in green light vs. red light as well as 17 genes not previously thought to be regulated by light [50]. Further exploration revealed a number of these genes had homologs in other organisms, though their functionality had not been fully deciphered [50]. In another study, both microarrays and proteomics were used to evaluate an *Escherichia coli *mutant secreting more α-hemolysin (HlyA) than the parent strain [51]. The researchers found decreased levels of tRNA-synthetases in the mutant as compared to the parent strain [51]. Based on this information, the researchers designed a modified hlyA gene to reduce the rate of translation by incorporating rare codons leading to the same amino acid sequence [51]. When the parent strain was transformed with this modified hlyA gene, it secreted even more HlyA than the mutant [51]. In other words, the study indicated it was possible to engineer cells using an approach that combined genomics and proteomics.

## Conclusion

Microarrays are a powerful genomics tool, designed to illuminate differences in the expression of genes within cells. Despite being a relatively new technology, the scientific community has quickly adopted its use in a variety of fields including drug development, evolutionary biology, and disease characterization [1,52]. The strength of the technology rests on the several factors including: ease of use, availability of platforms and lower cost relative to other exploratory methods such as Northern blotting or Ribonuclease Protection Assay (RPA), implementation of statistical methods for detailed analysis, and most importantly a global view of a gene expression encompassing an entire genome.

As previously eluded to, the technological limitations associated with microarrays manifest themselves in terms of variability typically seen as systematic errors. Improvements in robotics, array fabrications, and continued genome sequencing can certainly address these issues, but not entirely remove them. This places limits on what microarray technology can achieve, although a comprehensive understanding of microarrays can help establish meaningful and reproducible data. An effort to: properly design the experiment, establish quality control steps such as checking RNA purity, analyze the data, and verify the results can also combat technological challenges [10,14,53]. In addition, archiving databases and files is a consideration often overlooked, though quite important in being able to return to data with new leads and directions for subsequent research or simply cross-compare with new data.

There are, of course, other limitations, inherently present that restrict the scope of microarray analysis just like any other tool. For example, microarrays only present a snapshot of the transcriptome which is continually changing and responding to cellular needs and signals. As such, microarrays only illuminate a part of what is going on inside a cell or a population of cells [3,6]. In addition, there does not necessarily have to be a tight correlation between the expression of a gene and the amount of translated protein. Therefore, differentially expressed genes may not translate into varying protein levels with functional implications [3]. Furthermore, the complexity of microarray analysis makes it exceedingly difficult to ascertain meaningful data with real biological significance without clearly defined goals or targets. An intricate aspect of genomic analysis is the interplay between genes or groups of genes (i.e. mechanisms) and that information is not easily deciphered using microarrays. And finally, the functionality of a gene cannot be determined solely using microarrays [2,3]. Indeed, other methods and experimental tools are needed to decipher the proteome, understand the varying interactions between genes and/or proteins, and develop a more complete picture of cellular behavior.

Ultimately, microarrays will continue to be used in a variety of research areas as more options in the design of custom arrays become available along with an increase in the assortment of species-specific arrays. Technological advancements may help bring down the cost as well as enhance reproducibility and reliability promoting the applicaton of microarrays in new and diverse fields. In the end, the questions raised by microarray results are often just as vital as the answers they produce; a key to expanding the role of any scientific method to encompass new fields.

## List of Abbreviations

cDNA – complementary DNA

PCR – polymerase chain reaction

cRNA – complementary RNA

ESTs – expressed sequence tags

MGED – Microarray Gene Expression Data

EBI – European Bioinformatics Institute

MIAME – Minimum Information About a Microarray Experiment

GEO – Gene Expression Omnibus

NCBI – National Center for Biotechnology Information

TIGR – The Institue for Genomic Research

SOMs – Self-organizing maps

PCA – Principle component analysis

rRNA – ribosomal RNA

SNPs – single nucleotide polymorphisms

RT-PCR – reverse transcription-polymerase chain reaction

RPA – Ribonuclease Protection Assay

## Competing interests

The authors declare that they have no competing interests.

## Authors' contributions

PJ formulated the content, performed the literature search, and drafted much of the manuscript. KK contributed to revising the manuscript and adding content. MB contributed to formulating the content and layout. JS contributed to formulating the content, revising the manuscript, and drafting portions of the manuscript.
